# Toroidal electromagnetically induced transparency based meta-surfaces and its applications

**DOI:** 10.1016/j.isci.2021.103708

**Published:** 2021-12-29

**Authors:** Angana Bhattacharya, Rakesh Sarkar, Gagan Kumar

**Affiliations:** 1 Department of Physics, Indian Institute of Technology Guwahati, Guwahati 781039, Assam, India

**Keywords:** Applied sciences, Engineering, Photonics

## Abstract

The vigorous research on low-loss photonic devices has brought significance to a new kind of electromagnetic excitation, known as toroidal resonances. Toroidal excitation, possessing high-quality factor and narrow linewidth of the resonances, has found profound applications in metamaterial (MM) devices. By the coupling of toroidal dipolar resonance to traditional electric/magnetic resonances, a metamaterial analogue of electromagnetically induced transparency effect (EIT) has been developed. Toroidal induced EIT has demonstrated intriguing properties including steep linear dispersion in transparency windows, often leading to elevated group refractive index in the material. This review summarizes the brief history and properties of the toroidal resonance, its identification in metamaterials, and their applications. Further, numerous theoretical and experimental demonstrations of single and multiband EIT effects in toroidal-dipole-based metamaterials and its applications are discussed. The study of toroidal-based EIT has numerous potential applications in the development of biomolecular sensing, slow light systems, switches, and refractive index sensing.

## Toroidal excitations in MMs

**Figure 1:**
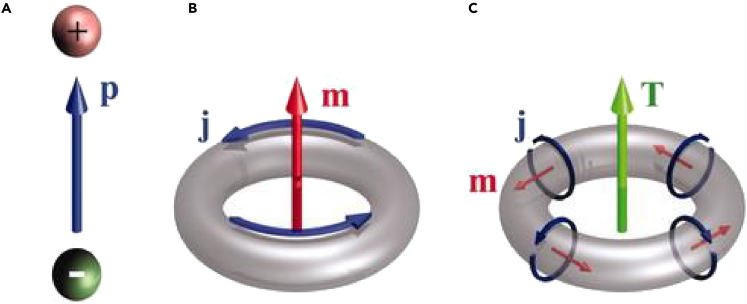
The formation of electric, magnetic, and toroidal dipolar moments (A) Electric dipolar moment is formed because of charge separation. (B) Magnetic moment formation because of the flow of current along a circular loop. (C) Toroidal moment formation because of the poloidal currents flowing along the arms of a torus. From (Kaelberer et al., 2010). Reprinted with permission from The American Association for the Advancement of Science.

Toroidal moments are a separate family of electromagnetic moments with properties different from the widely known electric and magnetic moments. They are not easily identifiable in natural materials as the contribution of the other electromagnetic moments masks their contribution. Although electric dipolar moment forms because of charge separation and magnetic dipole moment forms by current flowing along a circular loop, toroidal dipolar moment forms because of poloidal current flowing along the arms of the torus. The poloidal currents form magnetic moments aligned in a head-to-tail arrangement which leads to the formation of a toroidal dipolar moment along the symmetry axis of the torus (Kaelberer et al., 2010). Figure 1 illustrates the formation of toroidal moments. It clearly shows how the formation of current loops on a torus results in the formation of magnetic moments. These aligned magnetic moments further lead to the excitation of toroidal multipoles. Toroidal moments were initially introduced by ZelDovich in the context of nuclear and particle physics (Zel’Dovich, 1958). Toroidal excitations are identified as polar toroidal moments and axial toroidal moments. A ring of static or dynamic magnetic moments results in the excitation of the polar toroidal moment or a magnetic toroidal moment (Spaldin et al., 2008; Zimmermann et al., 2014; Fedotov et al., 2013; Fan et al., 2013). In contrast, axial toroidal moments are formed by a ring of electric dipolar moments (Dubovik et al., 1986; Thorner et al., 2014).

**Figure 2:**
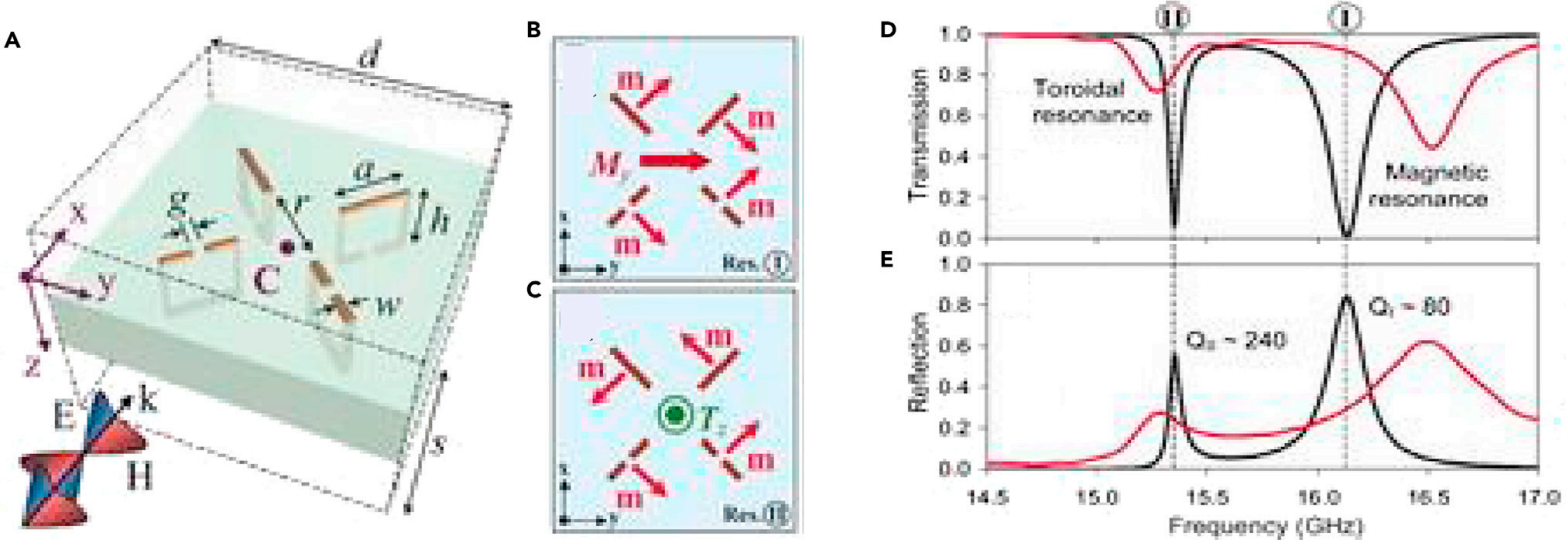
Experimental demonstration of toroidal excitation in metamaterials (A) Experimental setup of toroidal excitation achieved in the gigahertz regime for a MM design consisting of 3D loops of resonators. (B) Arrangement of magnetic moments ‘m'at resonance termed as I. (C) Head-to-tail arrangement of magnetic moments at resonance II, leading to the excitation of toroidal dipole moment along the z-direction. (D) Transmission spectrum of the MM depicting the origins of the two resonances. (E) Reflection spectrum of the MM with calculated Q factors of the resonances. From (Kaelberer et al., 2010). Reprinted with permission from The American Association for the Advancement of Science.

Because of the difficulty in observing toroidal excitations in natural media, metamaterials (MMs) have become significant in their study. Metamaterials (MMs) are artificially engineered materials consisting of periodically arranged subwavelength resonant unit cells known as meta-atoms. The properties of the MM do not depend on the intrinsic materials out of which it is made, but on the structural geometry of the constituent meta-atoms (Chen et al., 2006; Smith et al., 2004; Fan and Padilla, 2015; Cai and Shalaev, 2010; Rao et al., 2018). MMs have shown properties not seen in natural media including negative refractive index, superlensing, and artificial magnetism among others (Smith et al., 2004; Wong et al., 2017; Cai et al., 2007). MMs allow the manipulation of electromagnetic radiation for application-based photonic devices. Specially designed MM geometries ensured the dominating contribution from toroidal moments over electric and magnetic moment. Considering its potential in the design of photonic devices, toroidal excitations have found increasing applications in MMs (Marinov et al., 2007; Papasimakis et al., 2016). Such toroidal moments have been studied to show high Q factors and low radiation losses (Savinov et al., 2014; Talebi et al., 2018). The dynamic toroidal moments, which are induced by oscillating electromagnetic fields, represent the scattered power of radial currents. The electric and magnetic moments, in turn, are associated with transverse currents. Via the Taylor expansion of charge and current distribution, the toroidal dipole moment was described by the formula $T=1/10c\int d^{3}r\left[r\left(r.J\right)-2r^{2}J\right]\text{,}$ where *J* is the current density, *c* is the speed of light, and *r* is the radius of the toroidal geometry. The scattering of electromagnetic waves by toroidal dipole excitation scales as $∼R/\lambda^{3}\text{,}$ with λ being the wavelength of light and R being the length scale of the molecule (Papasimakis et al., 2016; Savinov et al., 2014). On the other hand, the scattered power of electric and magnetic moments scale as $R/\lambda \;\text{and}\;R/\lambda^{2}\text{,}$ respectively. The toroidal dipole interacts with external conduction and displacement currents, with the interaction energy given by $E_{T}=-T.\left(4\pi /c\;J_{ext}+1/c\;\partial D/\partial t\right)$, where *E*_*T*_ is the interaction energy of toroidal dipole moment, *J*_*ext*_ is the external conduction current and *D* is the displacement current (Papasimakis et al., 2016). The toroidal MMs are specifically designed by mimicking the torus configuration, such that the toroidal excitation gets amplified. To excite the carefully structured geometry of the toroidal MM, the light impinging on the MM may be incident normally or laterally. The polarization of the electric field of the incident light excites flow of circular surface current loops, ensuring the excitation of magnetic moments which, in turn, results in toroidal dipolar resonance. Because the far-field contribution of the toroidal excitation is similar to electric dipolar moment, destructive interference between the two leads to an anapole non-radiating configuration (Basharin et al., 2017). The net emission from a dynamic anapole being zero, it has emerged as a prominent area of research. The anapoles are not detected by far field radiation because of non-radiating nature. They can be detected when there is a slight imbalance in the destructive interference between toroidal and electric dipole resonances (Savinov et al., 2019). Considerable research interest has been put towards the field of anapole moment and its applications in recent times. Room temperature lasing from a metasurface composed of split nanodisc resonators supporting optical anapole states has been reported (Tripathi et al., 2021). Detailed review on the theory of the anapole mode, as well as experimental findings, has also been reported (Baryshnikova et al., 2019). Further, studies have been made on the electric and magnetic anapole mode and their properties (Luk'yanchuk et al., 2017). Similar to the high Q factor resonances of toroidal excitation, Fano resonances also show very high quality factors. The interference between continuum of states and quasibound states result in the asymmetric fano resonance. Studies have been made recently that reports generation of multipolar Fano resonance via the interference of bright dipole modes and dark multipole (Chen et al., 2017). Circular currents distribution forms an important parameter in determining toroidal excitation in metasurfaces. A recent study has demonstrated the excitation of an artificial transverse magnetic moment in a single plasmonic wire (Karmakar et al., 2021). The MM geometry consists of two unequal cutwires, and it is observed that increasing asymmetry in the geometry leads to a higher order mode which exhibits circular current flow in the longer wire. Thus, a dominant transverse magnetic signature is established in the plasmonic wire. Other recent studies have also reported high Q toroidal resonances by the near field coupling between neighboring magnetic moments (Kumar et al., 2020). Studies derived from fano-resonance based MM designs could be utilized in visualizing high Q toroidal geometries (Karmakar et al., 2020). Such studies could have significant potential in the design of circular current flow based plasmonic toroidal MMs. The first experimental study of toroidal excitations in MMs was demonstrated by Kelberer et al. in 2010. They designed 3D split-ring resonators in the form of loops, aligned such that magnetic moments generated were arranged end-to-end leading to the formation of toroidal dipolar excitation (Kaelberer et al., 2010). For z polarization of the incident beam, the electric dipolar moments were suppressed.The transmission spectrum showed two modes, termed as resonance I and II. At resonance I, it was observed that all the magnetic moments point along the same direction signifying the magnetic origins of the resonance. The second resonance, named resonance II, depicted the head-to-tail formation of magnetic moments indicating it's toroidal nature. The MM design is shown in Figure 2A. Figure 2B depicts the induced magnetic moments arranged such that a nonzero magnetic moment ‘$M_{y}$’is produced. Figure 2C shows the alignment of the magnetic moments in an anti-clockwise head-to-tail arrangement. This arrangement of the magnetic moments leads to the excitation of toroidal dipolar excitation along the z-direction (*T*_*Z*_). Figure 2D shows the transmission spectrum of the MM geometry, whereas Figure 2E shows the reflection spectrum along with the calculated Q factors of the resonances. The toroidal resonance was shown to display a very high-quality factor (Q) of 240. These experimental findings encouraged further research on toroidal MMs.

However, the fabrication of 3D MMs, carefully designed for a dominating toroidal response, is a challenging and time-consuming task. For ease of fabrication via photolithography and electron beam lithography, two-dimensional planar toroidal MMs have been examined in gigahertz, optical, and terahertz frequency ranges (Wang et al., 2018; Gupta and Singh, 2020; Fan et al., 2018; Huang et al., 2012; Ahmadivand et al., 2020; Guo et al., 2021; Tasolamprou et al., 2020; Chen and Fan, 2019; Stenishchev and Basharin, 2017). Significant contributions to toroidal moments have been made including the design of graphene-based actively tunable toroidal MMs, their applications in sensor, modulator designs, biological-based applications, and all-dielectric toroidal MMs (Bhattacharya et al., 2019, 2020, 2021). The next section discusses toroidal excitation in planar metasurfaces and in certain applications. We also deliberate on the identification of toroidal excitations in 2D planar MMs via surface current profiles, magnetic field profiles, and multipolar analysis.

## Toroidal excitations in planar meta-surfaces and their applications

**Figure 3:**
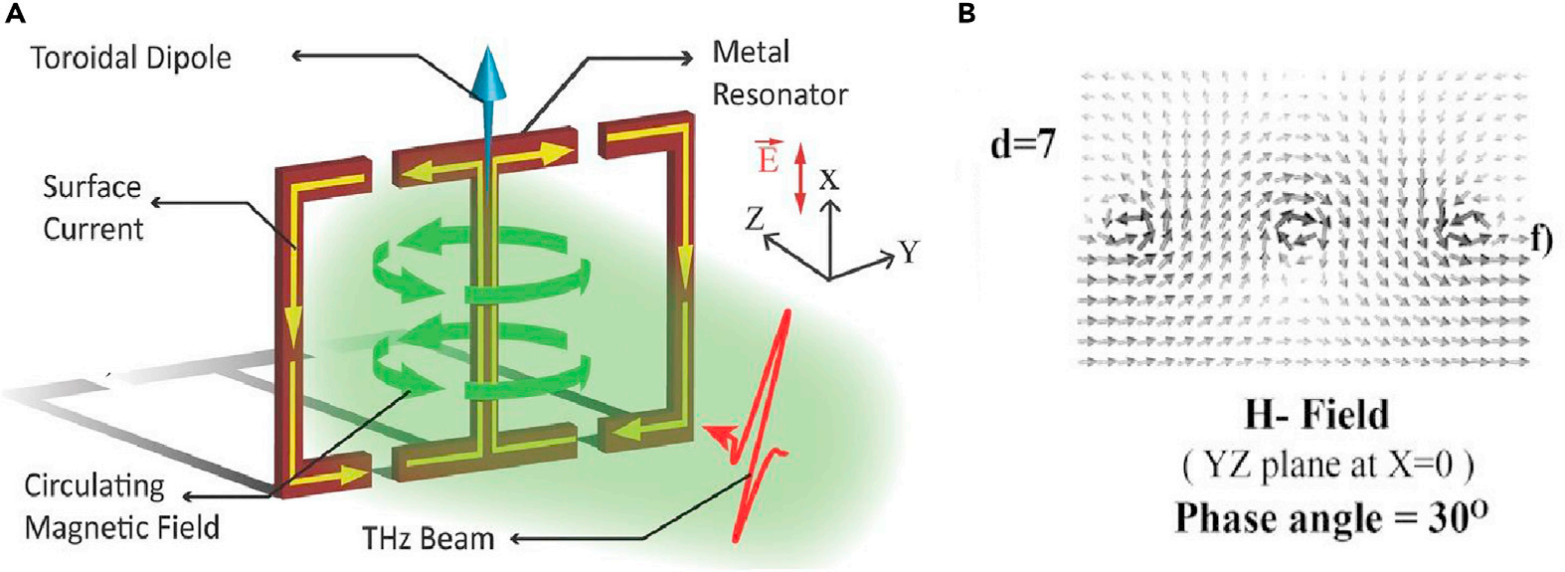
Toroidal resonance in a planar metamaterial geometry (A) Planar MM geometry exhibiting toroidal excitation via oppositely circulating current in the SRR arms. The orientation of surface current excites toroidal dipole moment along the blue arrow in the plane of the geometry. (B) Head-to-tail orientation of magnetic dipole moments indicating toroidal signature. Adapted with permission from ref (Gupta et al., 2016), John Wiley and Sons.

The complexity involved in the fabrication of three-dimensional metamaterials by conventional photolithography and electron beam lithography led to the exploration of planar toroidal MM designs. By analyzing the surface current profiles, magnetic field profiles, and electric field profiles for the fabricated planar geometry, it is decided whether the MM displays toroidal behaviour or not. Because the fingerprint for toroidal excitation is head-to-tail formation of magnetic moments, a cross-section of the design is often studied via some commercially available simulation software where the arrangement of magnetic dipoles becomes clearly visible. Similarly, the surface current profile is another significant tool in identifying the toroidal excitation. The direction of flow of current is analyzed to identify toroidal resonances. As discussed, the ease of fabrication of planar MMs over 3-dimensional structures has led to increasing research on planar meta-surface-based toroidal MMs. In this direction, Gupta et al. designed a simple toroidal planar MM geometry consisting of two joint double capacitive-gapped metal loops (Gupta et al., 2016). By carefully choosing the geometry, other electromagnetic moments are suppressed to ensure a higher contribution of the toroidal moment. Figure 3 shows the planar MM toroidal geometry which was studied.

Here a resonant mode was excited in the terahertz frequency range in which the current in the two loops oscillated in the opposite direction resulting in a toroidal dipolar response along the x-direction. The planar geometry ensured no component of toroidal moment T along the z-direction. The position of the gaps was varied to study the variation of the Q factor and to find the optimum configuration. Analysis of magnetic field profile was crucial in determining the predominance of the toroidal behavior. Figure 3B shows the magnetic moments arranged in a head-to-tail manner as was expected in the study. As previously discussed, such magnetic field profiles are indispensable in determining toroidal behavior (Kaelberer et al., 2010; Wang et al., 2018; Bhattacharya et al., 2020; Ahmadivand et al., 2017; Gupta et al., 2018).

**Figure 4:**
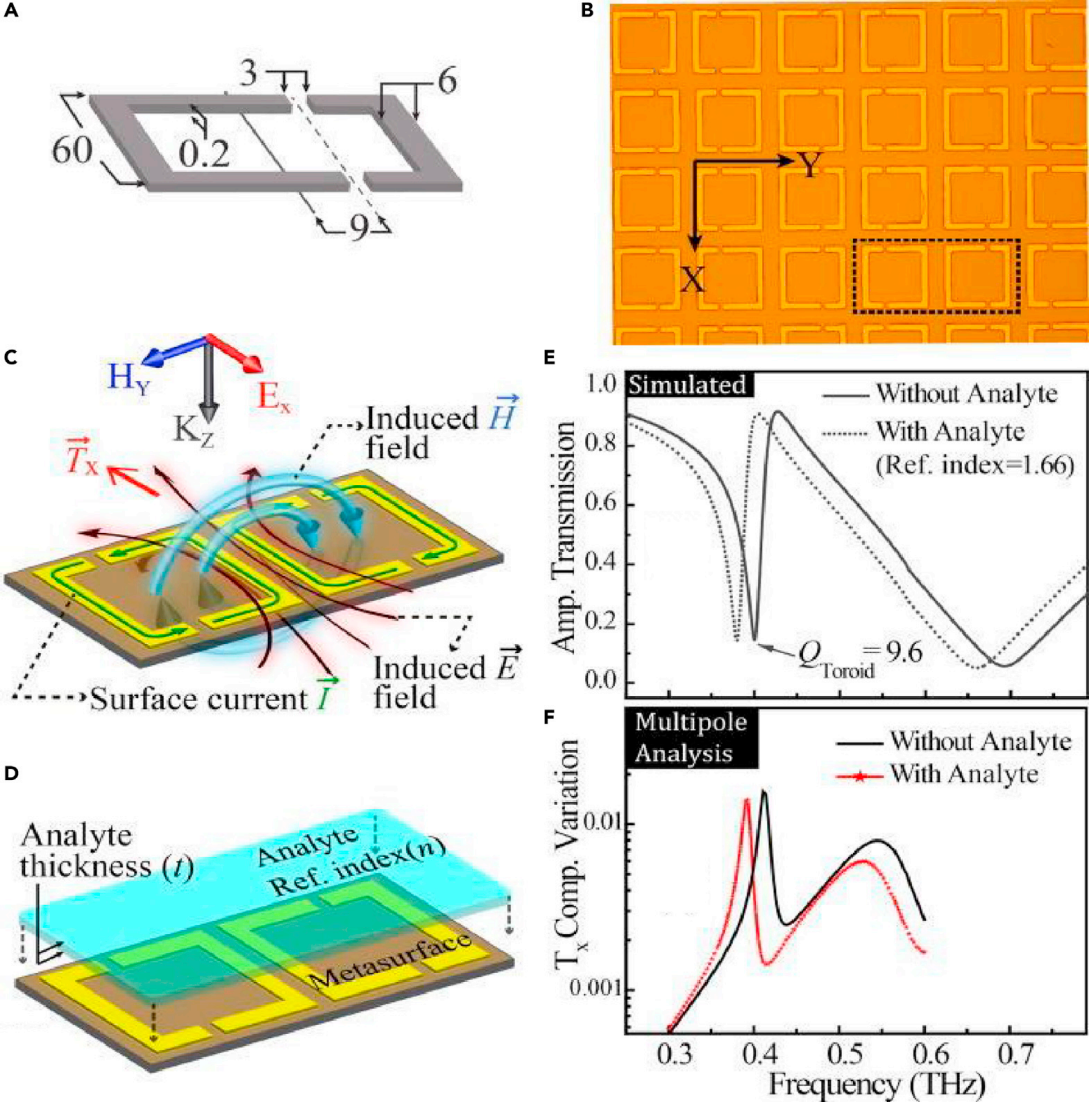
Refractive index sensing via a planar toroidal MM (A) Schematic of the MM. (B) The fabricated sample of the MM. (C) The surface current profile of the MM geometry. (D) Analyte of thickness ‘t'placed over the MM sample for refractive index sensing. (E) The transmission spectrum shows a red-shift on placing a layer of analyte (photoresist) over the two-dimensional toroidal MM. (F)The shift in resonance for *T*_*x*_ component of toroidal moment. Reproduced from (Gupta et al., 2017), with the permission of AIP Publishing.

The application of planar toroidal MM devices has been widely explored in recent times. Sensing using a terahertz toroidal 2-dimensional MM was investigated in a pair of mirrored asymmetric resonators (Gupta et al., 2017). A photoresist layer of refractive index 1.66 was coated on top of the resonators for varying thickness of the analyte. A red-shift in the toroidal dipolar resonance was clearly observed as shown in Figure 4. For different thicknesses of the photoresist, the redshift of the resonance was reported. Sensitivity calculations for different analyte thickness were observed and it was reported that the sensitivity increases with increasing analyte thickness and finally saturates. The sensitivity was reported to decrease exponentially on increasing the substrate thickness. The study indicated that toroidal resonances offer better sensitivity at large asymmetries of the resonators. The sensing of polar liquid analytes using a dual-torus metasurface has been reported recently (Xu et al., 2021). The study deals with the sensing of polar liquids by constructing a microfluidic channel through which the liquid is inserted. Refractive index sensing of the liquids is undertaken via the toroidal metasurface.

**Figure 5:**
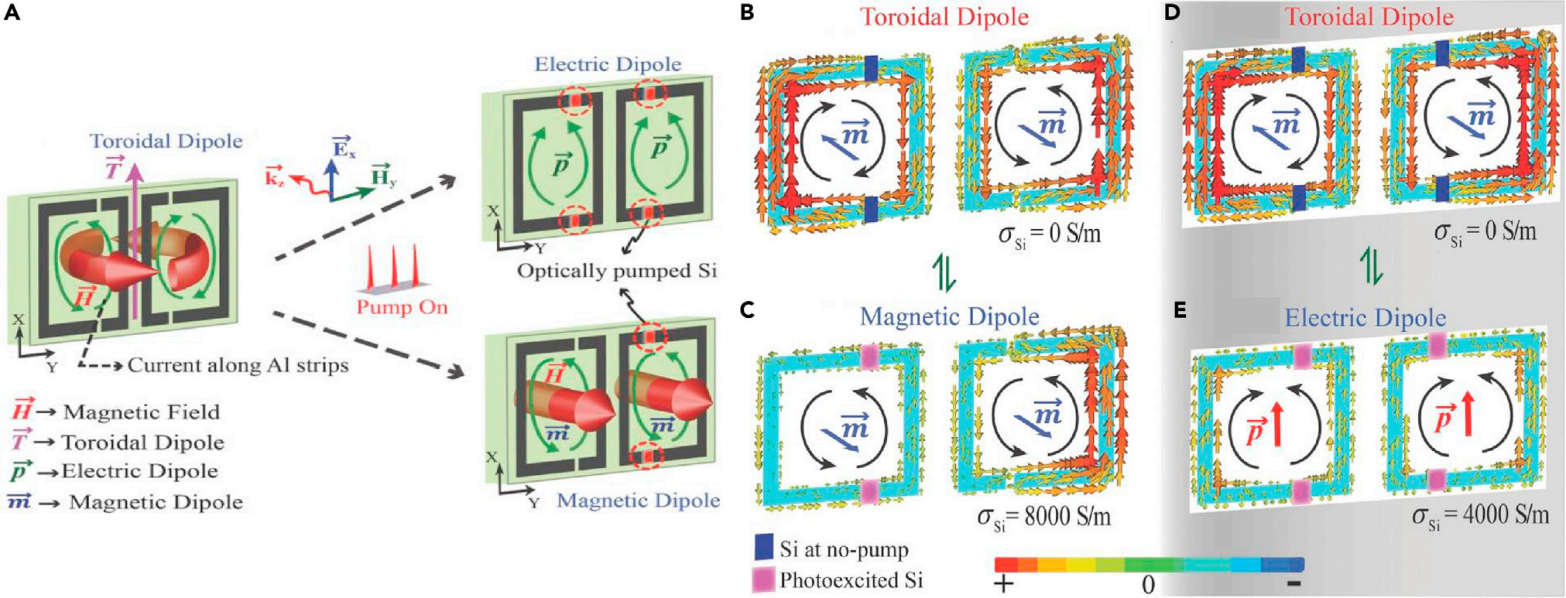
Application of toroidal excitation in a metamaterial based switch (A) Dynamic switching from toroidal dipole mode in the MM to magnetic and electric dipole mode via photoexcitation of silicon pads embedded in capacitive gaps. (B) Surface current profiles of the planar MM geometry showing alignment of magnetic moments in opposite directions in toroidal dipolar configuration. (C) Magnetic moments aligned along the same direction, indicating switching behavior from toroidal to magnetic mode. (D) Surface current profile for toroidal mode in the MM configuration where Si pads are inserted in both gaps. (E) Switching from toroidal mode to electric dipolar mode, depicted via surface current profile. Adapted with permission from ref (Gupta et al., 2018)., John Wiley and Sons.

**Figure 6:**
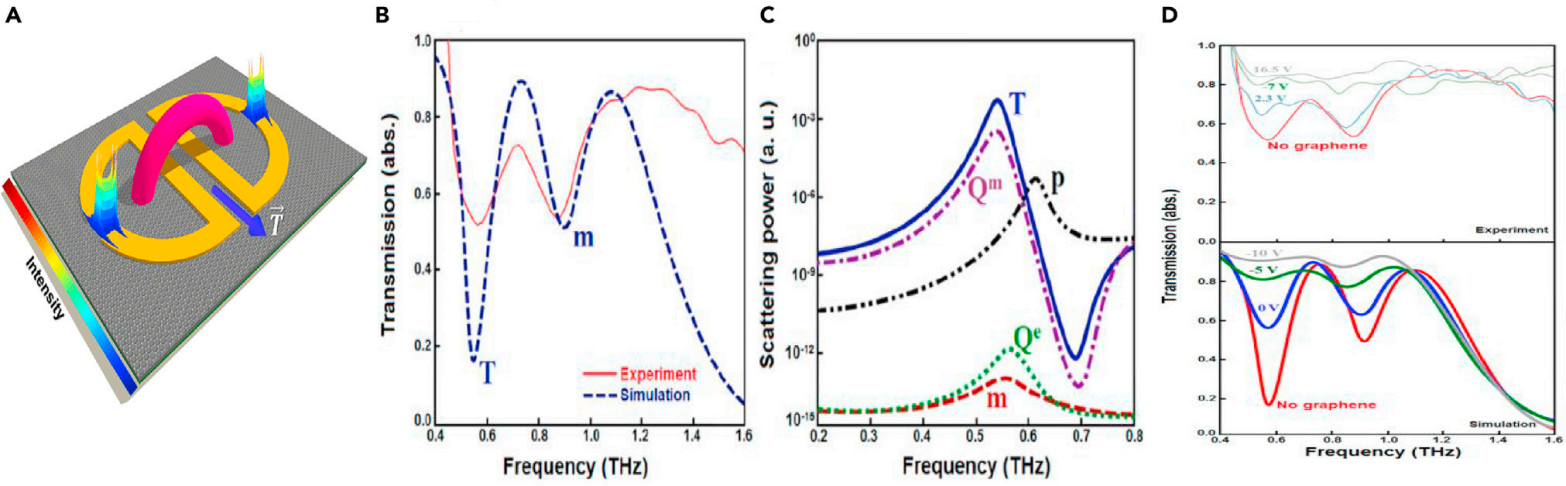
Active tuning of toroidal excitation in a terahertz metamaterial (A) Schematic of the proposed MM. (B) Transmission profile of the MM derived experimentally and via simulation. (C) The multipolar analysis evaluates the power scattered by the five major electromagnetic moments. Toroidal power scattered dominates over scattered power of other electromagnetic moments. (D) Experimental and numerical tuning of the transmission amplitude by varying the AC conductivity of graphene. Reprinted (adapted) with permission from (Ahmadivand et al., 2019). Copyright 2019 American Chemical Society.

Toroidal MM as a switch in the terahertz spectrum is of significant interest considering its numerous prospective applications. A study was conducted where a toroidal resonance was dynamically switched to an electric dipole resonance or a magnetic dipole resonance. The MM consisted of mirrored metal resonators with silicon (Si) pads layered beneath the capacitive gaps (Gupta et al., 2018). The MM geometry is shown in Figure 5A. When the meta-atoms were illuminated by incident THz radiation, a low frequency toroidal resonance and a high frequency electric dipolar resonance were observed. The sample was then illuminated by an optical pump beam in the presence of THz radiation. The photoexcitation of Si led to increased conductivity of the resonators. Two configurations were studied, one consisting of Si pads on the capacitive gaps of both the mirrored resonators. The other configuration consisted of Si pads on the gap of a single resonator. On increasing the pump fluence, it was observed that the toroidal resonance is killed and only the electric dipolar resonance remains for the first configuration. For high values of pump fluence, the strength of the magnetic dipole increased significantly in the second configuration. Thus, a dynamic switching behaviour from toroidal to electric and magnetic mode was reported as demonstrated in Figure 5A. Figures 5B and 5D show the surface current profile for the toroidal mode, where oppositely circulating current results in end-to-end formation of magnetic moments. Figure 5C depicts the surface current profile, whereby magnetic moments are aligned along the same direction, resulting in the magnetic mode. Similarly, Figure 5E shows the surface current profile indicating the electric dipolar mode. The switching from toroidal mode to magnetic dipolar mode is depicted in Figures 5B and 5C, whereas the switching from toroidal mode to electric dipolar mode is described in Figures 5D and 5E. The surface current is an important determinant for verifying toroidal behaviour in a MM sample (Basharin et al., 2017; Bhattacharya et al., 2021; Gupta et al., 2018; He et al., 2020). This study paved a path for new toroidal active devices in the THz regime. Besides passively modulated toroidal excitations, active toroidal MM devices have proved to be crucial in the development of high Q based photonic devices. This aspect of modulating toroidal resonances was explored by Ahmedivand et al. in a study where they designed a gate-controlled graphene mono-layered terahertz metamolecule, where the toroidal response was actively tuned by tuning the AC conductivity of graphene (Ahmadivand et al., 2019). The MM design is shown in figure 6A. The transmission spectrum for the geometry is shown in Figure 6B. By increasing the gate voltage of the p-doped graphene monolayer, the transmission amplitude was dramatically decreased. They further performed a multipolar analysis to evaluate the power scattered by electric, magnetic, and toroidal dipoles, as well as the contributions from the quadrupolar moments. This is illustrated in Figures 6C and 6D.

It was found that although the other multipoles were suppressed, the toroidal moment was significantly enhanced. Such an analysis reporting the contributions from different major electromagnetic multipoles has been a significant method for determining the domination of toroidal effects in a designed MM geometry. The scattered power contribution because of electric dipolar moment, magnetic moment, toroidal moment, and quadrupole moments are calculated over a range of frequencies by determining their individual contribution via surface current data (Kaelberer et al., 2010; Bhattacharya et al., 2021; Cong et al., 2017; Han et al., 2017). The study indicated the strong potential of the MM structure for use in active modulation and filtering purposes.

Although toroidal MM devices have made significant progress in recent times, the study of electromagnetically induced transparency (EIT) has also been widely investigated in MMs. The high Q and low radiation loss of toroidal resonances, combined with the exotic properties of EIT, creates a new field whereby the fascinating properties of both phenomena can be explored. In recent times, the excitation of EIT via interference with toroidal excitation has ignited considerable interest in the research community. Such designs could have implications in the development of EIT based photonic devices in the future. The next sections provide a brief discussion on the EIT phenomenon, its excitation based upon toroidal dipole resonances and potential applications.

## Electromagnetically induced transparency

Electromagnetically induced transparency (EIT) is the phenomenon where a highly opaque medium is rendered transparent to a probe beam at a resonance frequency in three-level atomic systems (Marangos, 1998; Boller et al., 1991; Fleischhauer et al., 2005; Kowalski et al., 1997; Harris, 1996). The destructive quantum interference between probe and pump beam explains the phenomenon. Research on EIT leads to several novel ideas including the slow light phenomenon and design of lasers without population inversion (Hau et al., 1999; Harris, 1989; Kash et al., 1999; Zibrov et al., 1995). In an effort to scrutinize the quantum effect in optical systems, the analogy of EIT in MMs caught the interest of scientists (Papasimakis et al., 2008; Liu et al., 2009; Zhang et al., 2008). The classical analogue of the EIT effect in MMs is often termed as plasmon induced transparency (PIT). EIT in MMs is often investigated via destructive coupling between ‘bright’ resonators and ‘dark’ resonators (Zhang et al., 2008; Devi et al., 2018; Singh et al., 2009). The bright resonators couple directly to incident light and are identified by low quality factors (Q) because of higher radiation scattering. The dark resonators demonstrate low Q factors as they are weakly coupled to incident radiation. The bright and dark mode resonators, with a similar resonant frequency, when brought together in a periodic MM arrangement, often display a narrow transparency band in an otherwise absorptive domain. This resonant transparency effect in the MM is known as EIT. The transparency window is often modulated actively or passively by changing the geometric parameters of the meta-atoms (Singh et al., 2009; Roy Chowdhury et al., 2013; Dhriti et al., 2021; Zhu et al., 2013; Dong et al., 2010). The EIT effect in MMs has raised significant interest over the years. The possibility of observing the effect at room temperature in MMs has also added to the interest. Split ring resonators (SRRs) coupled to cut wire (CW) configurations have been used to explain the EIT effect in the microwave, near-infra-red, optical, and terahertz frequency regimes (Dong et al., 2010; Liu et al., 2019). Well defined theoretical models have added strength to the experimental findings (Gu et al., 2012; Tassin et al., 2009). Dual band EIT, polarization independent EIT phenomenon, and slow light effect have been given considerable attention in recent years (Sarkar et al., 2019, 2020). EIT based MMs and their applications have seen increasing interest among the research community. Although most studies of EIT involve interference between dark and bright modes having electric or magnetic dipolar origin, the study of toroidal resonance based EIT has considerable relevance. The coupling between sharp toroidal excitation and dipolar resonance can lead to transparency windows possessing very high Q factors. Such aspects of coupling find use in sensing application in MM devices. The toroidal resonance having high Q is considered as a dark mode, whereas the broad electric dipole resonance is often considered as a bright mode. Numerous studies have been made in these areas reporting both single-band and multiband transparencies. The next section summarizes the investigations that have been made in this direction of toroidal excitation based EIT phenomenon.

## Excitation of toroidal dipole based EIT

The EIT effect in toroidal MM-based structures has caught the attention of the research community in recent years. Although the idea of toroidal excitation-based EIT is fairly new, its prospects in producing low loss MM devices have received considerable interest. Transparency windows have been observed via the coupling of high Q toroidal resonance to electric or magnetic resonance. The interaction of the dark toroidal resonance to a bright dipolar resonance resulting in a transparency window has seen significant interest in the research community. Although dark-bright dipole mode has already been extensively explored, the coupling to a toroidal mode has an added advantage of high Q resonance dips for the MM geometry. In this context, coupling mechanisms involving bright-bright modes as well as bright-dark modes have been reported. In the GHz frequency regime, the destructive coupling between an I-shaped cut wire resonator (ICW) and a spiral toroidal resonator has demonstrated a single transparency window (JunáHe et al., 2017).

**Figure 7:**
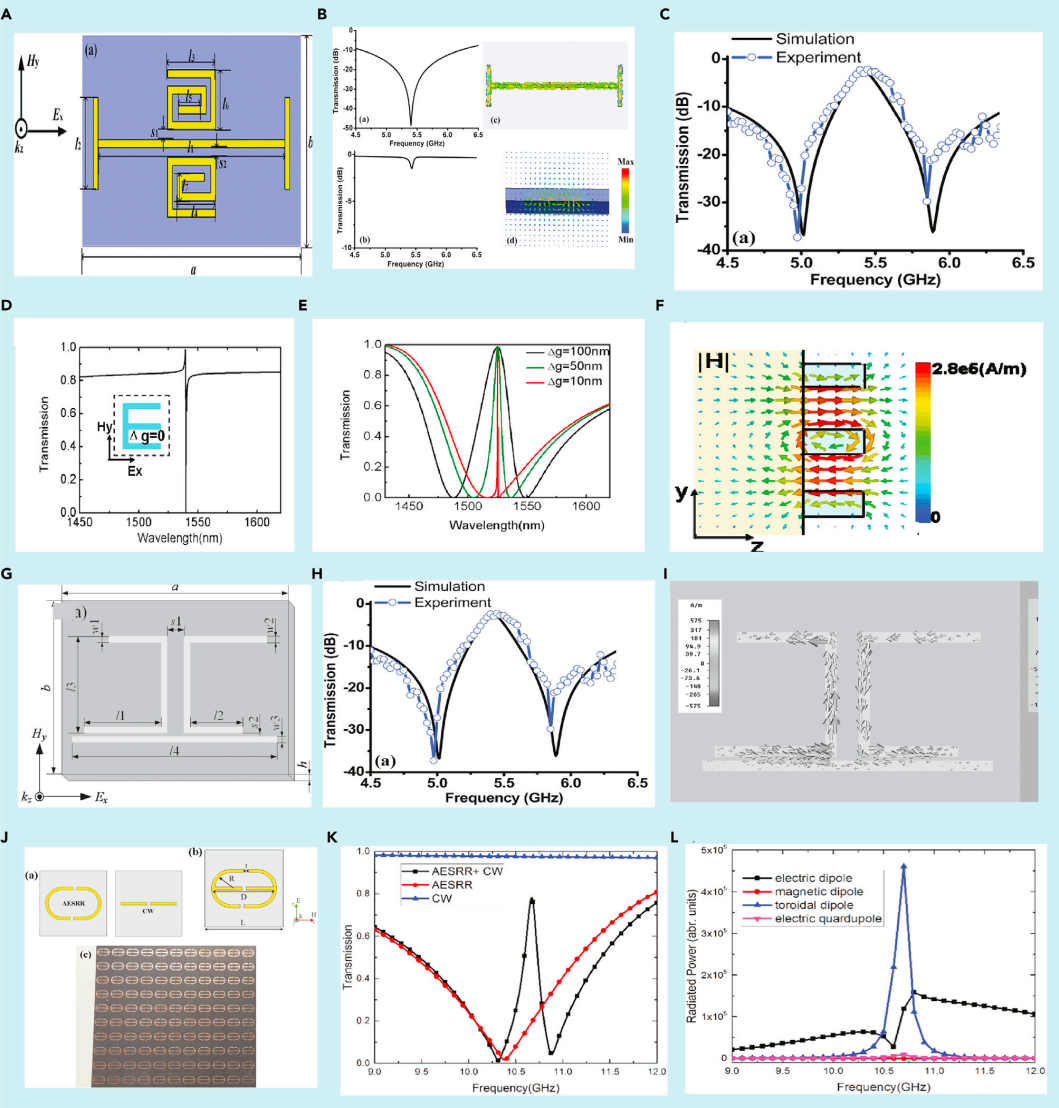
Toroidal excitation based EIT in MMs (A) Schematic of the toroidal based-EIT MM.b) Resonance frequencies of dark and bright mode with corresponding surface current profile and magnetic field profile. (B) The transmission profile of the MM array demonstrating single band EIT. (C) Simulated and experimentally obtained transmission spectrum for the combined MM geometry. (D) Fano resonance excitation for symmetric configuration of MM.Inset indicates MM geometry. (E) EIT response of transmission spectrum for increasing asymmetry. (F)Magnetic field profile of toroidal excitation. (G) Schematic for single band EIT MM. (H) Corresponding transmission profile of the single band EIT. (I) Surface current profile at EIT peak. (J) Schematic of asymmetric E type SRR and cut wire based MM geometry. (K) Excitation of single band EIT. l) Multipolar analysis of the corresponding geometry. Figures 7A–7C) Reproduced from Ref(JunáHe et al., 2017). with permission from the Royal Society of Chemistry, Figures 7D–7Ff). Adapted with permission from (Han et al., 2018)© The Optical Society, Figures 7G–7I). Reprinted from (Li et al., 2015), with the permission of AIP Publishing., Figures 7J–7L) Adapted from (Shen et al., 2020) with full permission.

The MM geometry is shown in Figure 7A. They utilized the dark-bright mode coupling between toroidal and electric dipole resonance to highlight the EIT phenomenon. When an electric field polarized along the x direction is incident on the MM, circular currents are excited in the spiral SRRs leading to a toroidal dipole excitation. Further, the excited toroidal dipole resonance is pointed parallel to the electric field which verifies its electric dipole origin. The toroidal spiral SRR acted as the dark mode, whereas the electric dipolar excitation from the ICW acted as the bright mode. The magnetic field profile of the spiral split ring resonators indicated their toroidal behaviour, whereas the surface current profile of the cut-wire indicates its electric dipolar nature. A broad resonance at 5.4 GHz, having a small quality factor of 1.2, was observed from the transmission plot of the cut wire. Meanwhile, the spiral toroidal resonators show a small resonance dip at 5.4 GHz having a Q factor of 60.3. This can be observed from Figure 7B. The combination of both these resonators led to the EIT response in the MM as can be observed from Figure 7C. They also reported a slow light effect in their proposed geometry. The destructive interference between a dark toroidal mode and a bright magnetic dipolar moment leading to an EIT-like optical response has also been reported in the near-infrared region in an all-dielectric meta-surface (Han et al., 2018). The MM geometry can be observed from the inset of Figure 7D. For an electric field polarization along the y axis, a sharp fano like resonance is observed. However, the magnetic dipole moment is the highest for y polarized configuration. When the MM geometry is excited by an x polarized electric field, a broad resonance is observed for the symmetric design (Δ*g* = 0). By introducing an asymmetry parameter in an E-type silicon array (Δg≠0), the EIT window was introduced. It was observed that higher the asymmetry parameter, the broader was the EIT window. This decrease in the width of the EIT window on decreasing asymmetry is shown in Figure 7E. Further, the surface current profile at the resonance frequency indicated toroidal dipolar excitation as can be seen in Figure 7F. Similar work was reported indicating EIT by interference between dark toroidal mode and bright electric dipolar mode in the GHz region. The behavior of surface current confirms the EIT effect (Li et al., 2015).

In this study, the electric field presents along the x-direction excited a toroidal dipolar response in the resonators. Hence, it was termed as an electric toroidal dipolar response. This toroidal resonance had a high Q factor and was treated as a dark mode. Further, the cut wire acted as electric dipolar bright mode. When the toroidal SRRs and the cut-wire were assembled together in the MM configuration, as shown in Figure 7G, a transparency window was observed. The excited EIT window is shown in Figure 7H. The EIT window is excited with a resonance peak at 8.12 GHz. The surface current profiles and magnetic field profiles provided an affirmation that the destructive interference between the ASSRs and cut-wire led to the EIT window.

It is observed that for the resonance dip at 7.92 GHz, surface current is mostly excited along the cut wire and ASSRs. For the transmission dip at 8.47 THz, all the currents are directed along the cut wire. But at the EIT peak of 8.12 GHz, most currents are distributed along the ASSRs and much less along the SRRs indicating the destructive interference between the two which led to the excitation of the EIT window as can be observed from Figure 7I. A high Q factor EIT, with polarization sensitiveness in the GHz region, was investigated by the combination of an asymmetric SRR (AESRR) and a cut wire (CW) (Shen et al., 2020). The CW acted as a dark mode, whereas the AESRR acted as a bright mode. Figure 7J shows the MM geometry. Figure 7K illustrates the excitation of the EIT window because of the destructive interference between dark mode of CW and bright mode of AESRR. A multipolar analysis indicated the domination of toroidal dipolar excitation in the EIT window. This is shown in Figure 7L. The EIT phenomenon in toroidal MMs has been explored via different designs in the GHz range. It was reported that illuminating incident radiation perpendicular to the gaps, in an asymmetric toroidal SRR, led to an EIT window (Xiang et al., 2018).

**Figure 8:**
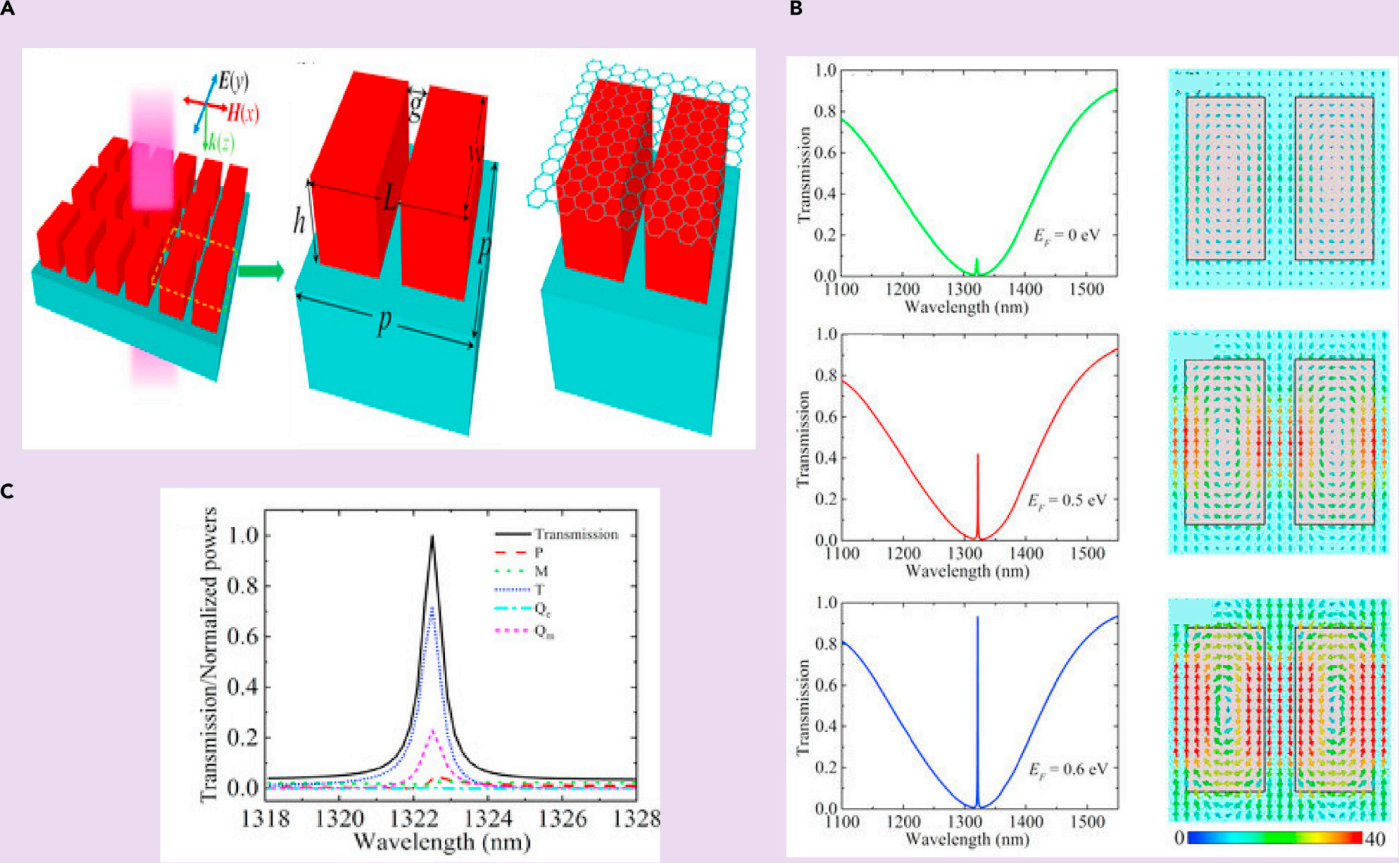
Actively tunable toroidal based EIT for an all dielectric metasurface made of silicon nano-cuboids with a active tuning enabled via a graphene layer (A) Schematic of the all dielectric metasurface. (B) Transmission spectrum for different values of Fermi energy. (C) Multipolar analysis of the MM geometry (Sun et al., 2020).

Recently, the dynamic manipulation of EIT in the optical range has been reported in a graphene based all dielectric metasurface (Sun et al., 2020). Figure 8A shows the metamaterial design of the all dielectric metasurface. It was shown numerically that the destructive interference between a toroidal mode and a magnetic dipolar resonance, in a metasurface made of split silicon Nano-cuboids, led to EIT. The active manipulation of toroidal dipole-based EIT via the exotic properties of graphene was explored in the study. Monolayer graphene placed over the metasurface enabled active tuning and manipulation of its transmission behavior. The transparency window showed a prominent transition as the Fermi energy was varied, acting as an optical switch. Figure 8B shows the modulation of transmission amplitude with increase in Fermi energy of graphene. Moreover, it is observed from Figure 8C that the multipole analysis shows domination by toroidal scattered power for the metasurface. Further, in the terahertz region, the coupling between two adjacent toroidal dipolar responses leading to EIT has also raised significant interest (Wang et al., 2021). In this work, two asymmetric J-shaped resonators were fabricated on a metasurface. The mutual coupling between the two resonators resulted in dual toroidal dipolar resonances in the terahertz regime. A red shift in the resonances was reported on increasing the length parameter of the meta-atom. Further, it was shown that breaking the symmetry in the structure resulted in the dual resonances. The surface current profiles and magnetic field distribution confirmed toroidal behavior of the resonances. It was found that the coupling interference between the two toroidal excitations results in the EIT effect. When the two resonances couple together, the destructive interference between them suppresses the broader resonance. This in turn leads to an induced transparency window. Hence, the study analyzed a tunable single band EIT based toroidal THz MM.

**Figure 9:**
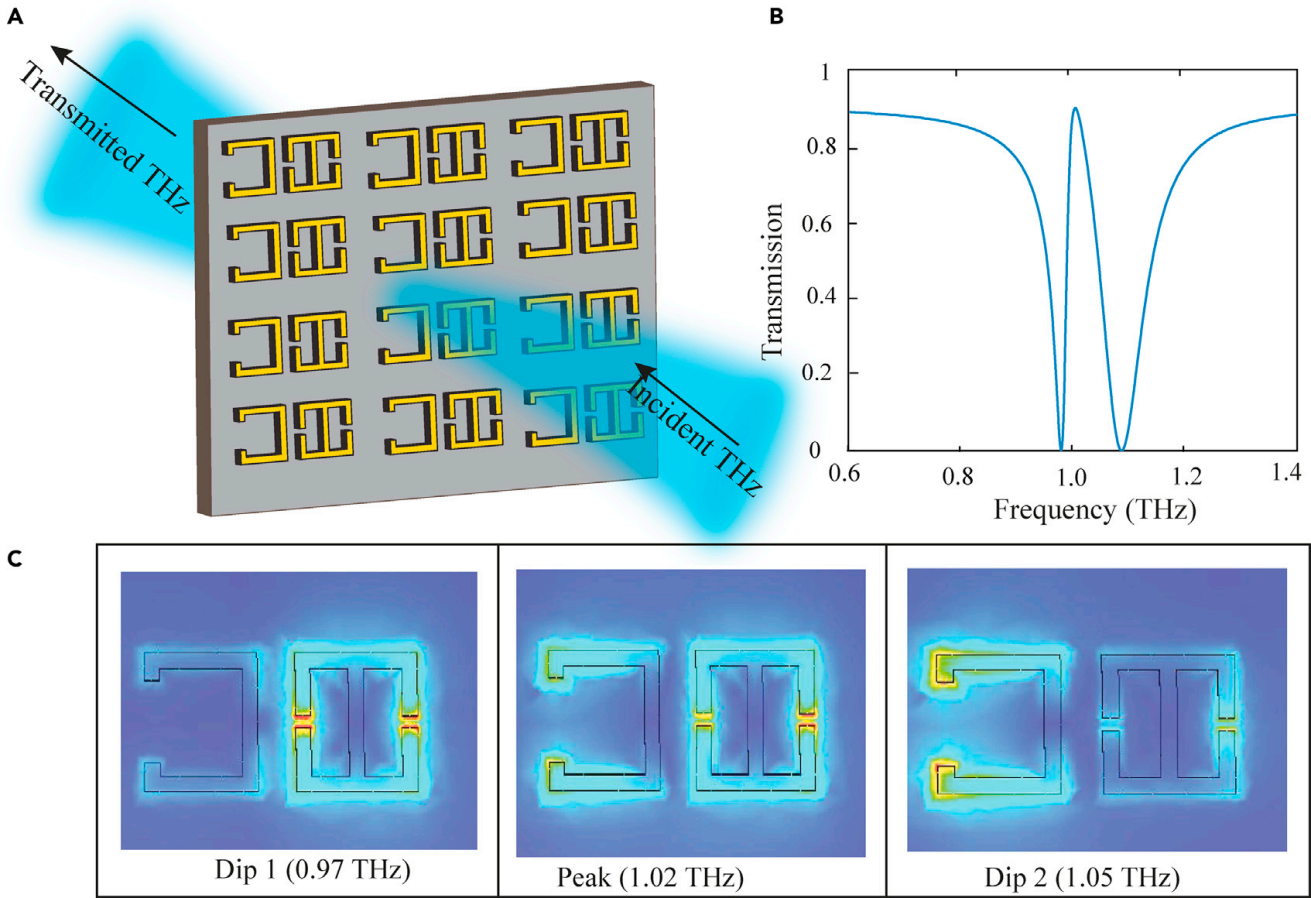
Demonstration of single band EIT in a toroidal terahertz metamaterial (A) Schematic of the toroidal based single-band EIT MM in the THz range. The incident THz field is polarized parallel to the split gap i.e., along y axis. (B) The transmission profile of the proposed MM array demonstrating single band EIT. (C) Electric field profiles at 0.97 THz (dip 1), at the peak frequency of 1.02 THz and at 1.05 THz (dip 2).

Based on this study on THz single band EIT, we designed a MM geometry which demonstrated coupling between bright toroidal-bright LC mode, resulting in a single band EIT window in the THz region. The MM configuration consisted of a double capacitive-gapped toroidal SRR and a C shaped dipolar resonator. This is illustrated in Figure 9. Figure 9A shows the designed MM array on which THz radiation is incident. The transmission profile of the MM array is shown in Figure 9B. It is observed that in between the dips corresponding to 0.97 THz (dip 1), and 1.05 THz (dip 2), a transparency window is evident with a peak at 1.02 THz. The CST simulations of the transmission profile for the double gapped resonator and the C resonator showed individual resonance frequencies at 0.976 THz and 1.05 THz, respectively. The electric field profiles in Figure 9C further validate this. It shows the excitation of the toroidal SRR at 0.97 THz, the excitation of the left CSRR at 1.05 THz, and at the peak frequency, both the resonators are excited. The excitation of both resonators at the transparency peak frequency in bright-bright mode coupling based EIT has been reported in literature (Yahiaoui et al., 2018). Hence, we demonstrated a single EIT window in the THz range via the bright-bright coupling of toroidal resonance to an LC resonance.

**Figure 10:**
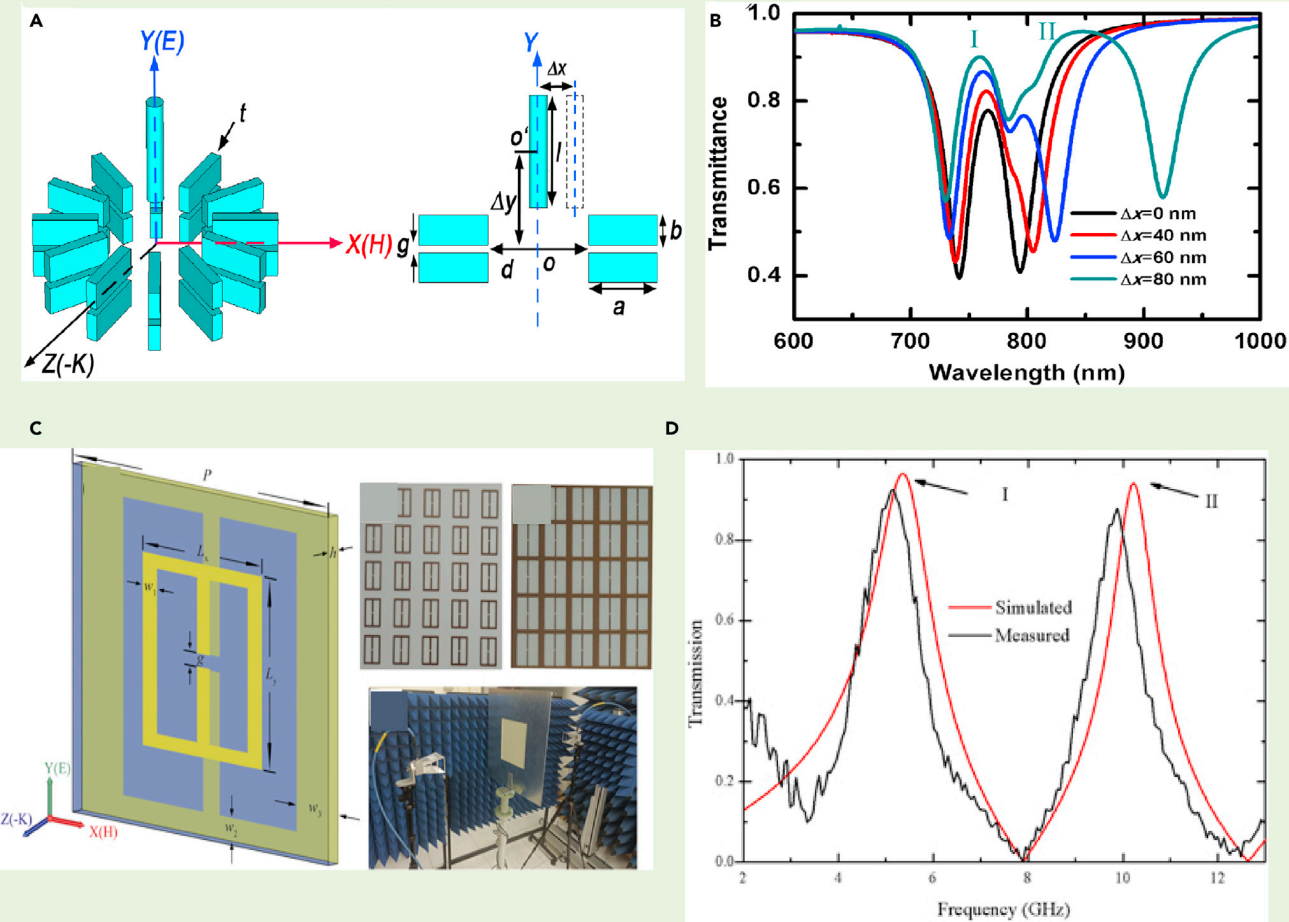
Multiband EIT in toroidal MM (A) Schematic of the 3D MM geometry consisting of 12-fold double metal bars and a rod demonstrating EIT in the optical range. (B) The corresponding transmission profile of the MM array for increasing asymmetry along the x direction. (C) Schematic of double sided toroidal planar MM demonstrating dual EIT in the microwave range. (D) Corresponding transmission profile of the MM geometry. Figures 10A and 10B. Reprinted from (Li et al., 2016), with the permission of AIP Publishing. Figures 10C and 10D (Lei et al., 2021) Copyright (2021) The Japan Society of Applied Physics

**Figure 11:**
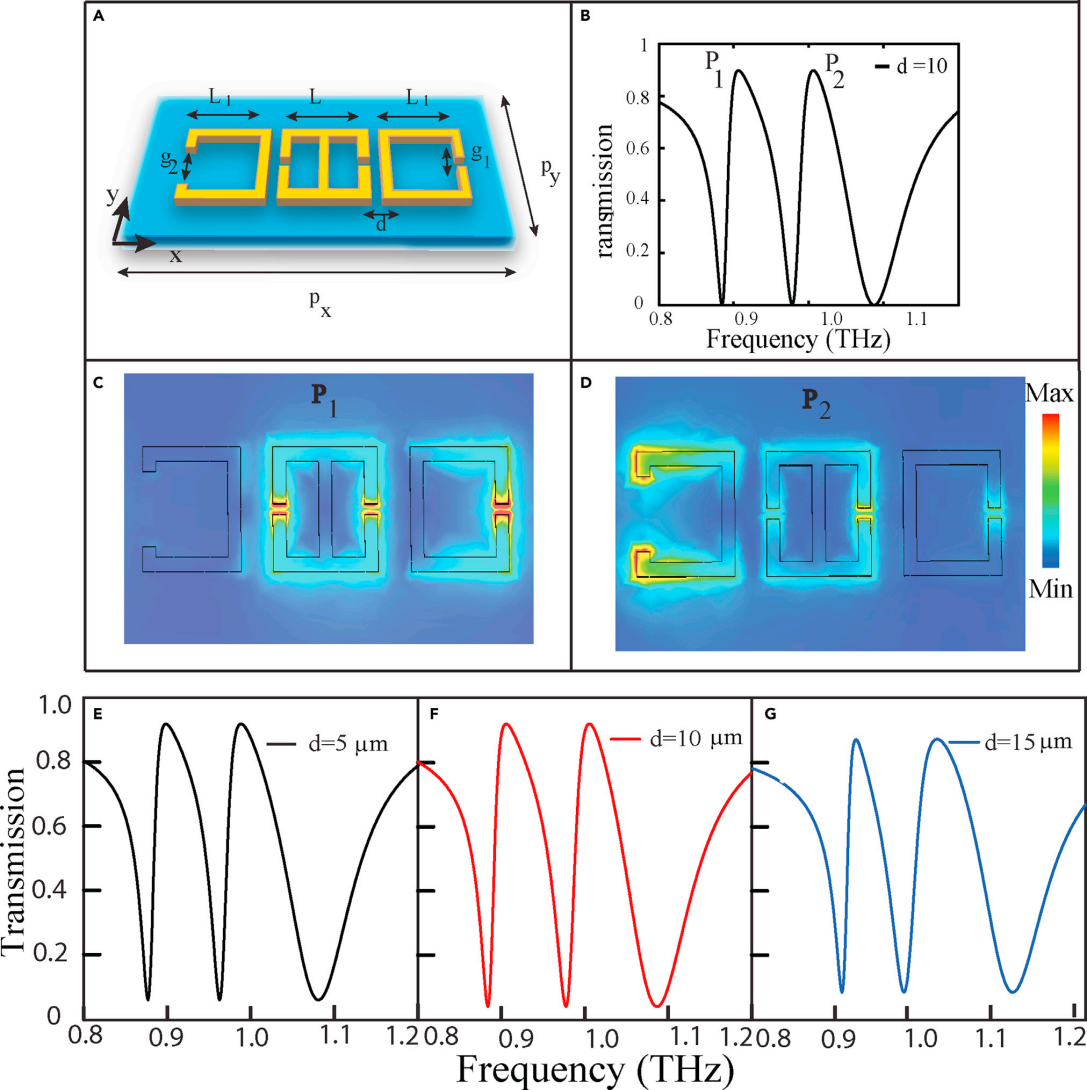
Multiband toroidal excitation based EIT in terahertz metamaterial (A) Schematic of the toroidal metamaterial design exhibiting multiband EIT phenomenon. The incident THz field is polarized parallel to the split gap i.e., along y axis. (B) Magnified view of the unit cell of the MM. ‘*P*_*x*_’, ‘*P*_*y*_’are the periodicities of the unit cell. Split gaps are denoted by ‘*g*_*1*_’and ‘*g*_*2*_’. The length of mid SRR (TSRR) is ‘L'and that of the other C shaped SRRs (CSRR) is ‘*L*_*1*_’.The distance of each CSRR from the mid TSRR is termed as ‘d’. (C) Transmission spectra showing multiband transparency effect for ‘d’ = 10 μm. (C) The electric field profile at the first peak *P*_*1*_. (D) The electric field profile at the second peak *P*_*2*_. Transmission spectrum for different values of distance ‘d’, which signifies the distance between adjacent resonators in our proposed MM geometry, i.e., d = 5 μm. (E), d = 10 μm. (F), and d = 15 μm. (G). A blue shift is observed on changing ‘d'from 5 μm to 15 μm which may be attributed to the reduced coupling between the resonators on increasing d (Bhattacharya et al., 2021).

Although single band EIT has found prominence in literature, recently the potential of EIT in multiband frequency regime has been paid significant attention by the research community. The advantage of multiband EIT over the single band EIT lies in the fact that one can manipulate the light-matter interaction over multiple windows in the frequency spectrum. Such investigations provide the flexibility in designing multiband sensors, modulators, and optical switch devices. In this context, a three-dimensional metamaterial geometry involving 12 fold double metal bars and an upright metal rod were designed to create an EIT window in the optical frequency range, by simultaneously exciting electric and toroidal excitations (Li et al., 2016). The geometry for the metamaterial is depicted in Figure 10A. The movement of the metal rod leads to an asymmetry whereby the second transparency window was observed. The coupling between the metal bar and each individual rod led to the excitation of the dual-band transparency window. This is illustrated in the transmission spectra of Figure 10B. It is observed that the transverse displacement of the metal rod leads to a dual-EIT like phenomenon. Further, in the microwave regime, a dual EIT in a planar toroidal MM design has been explored (Lei et al., 2021). In this study, a planar structure was designed with meta-atoms on both the front and back sides of the substrate. Figure 10C illustrates the MM geometry. When electromagnetic radiation was incident, parallel to the split gaps, the transmission spectra resulted in two transparency peaks. This was reported as a dual EIT phenomenon. The two transparency peaks that were excited are shown in Figure 10D. Surface current profiles and magnetic field profiles confirm that the peaks showed toroidal behavior. Scattered powers of different multipoles also verify the domination by toroidal excitation. Another study has shown dual-band EIT in a MM design which unites an E type and ‘ε’type resonator (Shen et al., 2020).The carefully designed MM geometry displays dual-band EIT. Although the first peak in the transmission spectrum of the EIT window demonstrated magnetic dipolar dominance, the second peak showed toroidal dipolar characteristics. The surface current profile and magnetic field profiles confirm the behavior. Refractive index sensing was further explored in the study. Literature shows that the field of dual EIT in toroidal dipolar-based MMs has ample scope of new research in the fast progressing field of terahertz MM photonic devices. Hence, a wide range of ideas can be explored in designing toroidal dipole-based EIT devices in the terahertz spectral regime. A study has been reported recently which reports multiband toroidal-EIT based excitation in the THz region. The MM geometry couples a toroidal resonance to a dipolar resonance in the THz spectrum resulting in a multiband transparency effect (Bhattacharya et al., 2021).The proposed geometry for multiband transparency effect in the THz regime consists of two C-shaped resonators (CSRRs) coupled to a double capacitive gapped split ring resonator. The two C resonators have different capacitive gaps. The design is illustrated in Figure 11A Here, the response of each individual resonator was investigated. The surface current profile for the mid resonator showed toroidal resonance behavior. The transmission plot of the combined MM geometry, as illustrated in Figure 11B showed two transparency windows in the THz frequency range. The electric field profile of the MM indicated the bright-bright mode coupling of the toroidal resonator to each C resonator at the peak frequencies. At the first peak frequency (*P*_*1*_), the coupling between the mid toroidal resonator (TSRR) with the right CSRR leads to the first transparency window. The coupling between the TSRR and left CSRR at the second peak frequency (*P*_*2*_) lead to the formation of the second transparency window. This coupling behaviour at the two peaks is evident from Figures 11C and D. The MM design could successfully demonstrate toroidal induced multiband transparency in the terahertz spectrum. Further studied the modulation of the transparency windows on increasing the distance d, i.e., the separation between the TSRR and the CSRRs. Figure 11E indicates the transmission spectrum for d = 5 μm, 11 (f) shows the transmission for d = 10 μm, and Figure 11G shows transmission for d = 15 μm. It was found that the transparency windows demonstrate a blue shift on increasing the value of d. The blue shift in the transmission spectrum as d is increased from 5 μm to 15 μm is observed in Figures 11E–11G. The blue shift in the transparency windows could be attributed to the reduced coupling between the resonators. A theoretical model has been developed in the study which confirmed the coupling behavior. The coupling parameters in the theoretical model decreased on increasing the distance between the resonators. This study of multiband excitation of toroidal EIT could be significant in fabricating low-loss toroidal photonic devices in the near future. The dual transparency windows allow the manipulation of light over multiple regions of the THz spectrum and, thereby, provide an added advantage to the investigation of EIT based phenomenon. The single band and multiband EIT has been widely explored, as discussed in the last section. The intriguing properties of EIT have been extremely fascinating to the research community in the last few decades. This attention towards EIT based devices has led to the wide investigation of its applications. The next section explores certain applications of the EIT phenomena for the development of photonic devices.

## Applications of toroidal EIT

### Slow light effect

A promising application of EIT is the slow light effect. In the slow light phenomenon, the group velocity of light passing through a medium reduces dramatically. The sharp transmission profile of the EIT window is associated with a steep linear dispersion within the transparency window, which results in a high group refractive index. This high group refractive index causes a dramatic reduction of group velocity, which has potential application in the storage of light and in optical buffer devices. In slow light metamaterials, photons can be trapped for a longer time, and hence light matter interactions can be increased significantly. The group delay can be calculated using the following formula,

(Equation 1) $$\tau_{g}=-\frac{d\varphi }{d\omega }\text{,}$$

where φ is the transmission phase shift. The corresponding group refractive index could be computed using the expression,

(Equation 2) $$n_{g}=\frac{c}{\nu_{g}}=\frac{c}{h}\tau_{g}=-\frac{c}{h}\frac{d\varphi }{d\omega }\text{,}$$

where *c* is the velocity of light in free space, *v*_*g*_ is the group velocity of light, and ‘h'is the effective thickness of the MM. The group refractive index computed using the given formula turns out to be very high within the transparency window.

**Figure 12:**
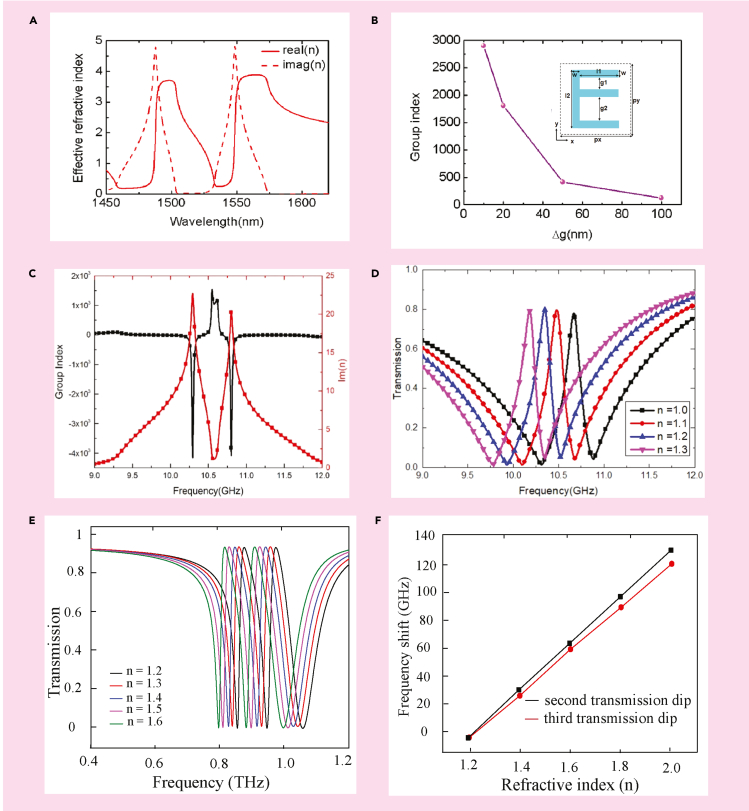
Applications of EIT based toroidal MMs (A) Variation of effective refractive index with wavelength for ’E’ shaped resonator. (B) Variation of group index with asymmetry parameter. (C) Variation of group index with frequency for a toroidal EIT MM in the GHz regime. (D) Sensing application of the MM showing transmission spectra for varying refractive index of the surrounding medium. (E) Numerically calculated transmission spectra for different refractive index (n) of analyte coated on the top of the proposed MM. (F) The shift in frequency for the second and third transmission dips with varying refractive index of the analyte. Figures 12A and B Adapted with permission from (Han et al., 2018)© The Optical Society. Figures 12C and D Adapted from (Shen et al., 2020) with full permission.

The slow light phenomenon was explored in an asymmetric E-shaped all dielectric toroidal meta-surface (Han et al., 2018). It was proposed in the near infrared frequency regime. For symmetric split gap, i.e., for Δ*g = g*_*2*_ *−* g_1_= 0, the structure shows a toroidal resonance at 1540 nm. A narrow transparency window was reported for their proposed structure for Δg≠0 . To study the slow light effect in their proposed geometry, they plotted the real and imaginary parts of the effective refractive index retrieved from the transmission and reflection spectra, for Δ*g* = 100 nm. The dip in the imaginary part signifies that the meta-surface shows a low-loss EIT effect. As Δ*g* reduces, the EIT window becomes sharper, which can be visualized by increasing the value of the group refractive index.Figure 12A shows the variation of the effective RI with wavelength, whereas Figure 12B illustrates the variation of the group index with change in parameter g. The inset picture indicates the MM geometry. Slow light phenomenon in toroidal EIT based MM has also been reported in another recent study (Shen et al., 2020). They reported the variation of group index and the imaginary part of RI with frequency. Figure 12C illustrates these results. It is observed that the group RI increases to 1537 at 10.67 GHz, whereas the imaginary part of RI at the corresponding frequency is almost 0. This indicates that the loss at the transparency peak is minimal. In the study discussed above, the modulation of EIT window and slow light effect was achieved passively by changing the structural parameters. The practical implementation of such structures becomes quite complex. For practical applications, it is highly desirable to achieve active control of the EIT window and slow light effect. Recently, graphene based MMs have attracted attention among researchers because of their exceptional optical and electronic properties. The conductivity of graphene can be modulated by changing the external bias voltage, and hence by changing its fermi energy. In this context, Sun et al. have reported dynamic modulation of slow light effect in a graphene loaded all-dielectric meta-surface (Sun et al., 2020).They further explored the active modulation of group refractive index and group delay. For *E*_*F*_ < 0.47 eV, the group delay is 1 ps but as the *E*_*F*_ increases beyond 0.6 eV, a maximum group delay of 3.1 ps is observed. On the other hand, a maximum of 3390 group index can be observed for *E*_*F*_ = 0.7 eV. Such studies prove to be significant in the quest for new ideas on the active modulation of toroidal-EIT responses.

### Refractive index sensing

Generally, the sensing capability of a device is characterized by high Q factor, high sensitivity, and the figure of merit (FOM). Refractive index sensing of a proposed meta-structure is usually examined by studying the shift in frequency of the transmission dips, or transparency peak (Δ*f*) with the change in the refractive index (n) of the analyte coated on top of the resonators. One can calculate the sensitivity (S) from the slope i.e., (Δ*f*/Δ*n* ) of the Δ*f* vs Δ*n* curve. On the other hand, the FOM is defined as FOM = Q × S = S/FWHM, where Q is the quality factor of the resonance and FWHM is the full width at half maximum of the resonance. As discussed earlier, the excitation of toroidal resonance in a planar structure is associated with a high Q factor and low loss. Hence EIT effect induced by toroidal-dipolar or toroidal-toroidal resonances could provide us a platform for better sensing applications. In this context, Shen et al. investigated a planar EIT MM composed of an asymmetric ellipse split ring resonator and a cut-wire numerically and experimentally (Shen et al., 2020). The proposed structure exhibits a strong toroidal dipole resonance at the transparency peak at 10.67 GHz. The sensing capability of their proposed MM was investigated by plotting the transmission spectra of the structure versus the change in refractive index of the surrounding media. The refractive index varied from 1.0 to 1.3. As the refractive index changes, the transparency window gets red shifted. A sensitivity of 1.9 GHZ/RIU and a FOM of 13.38 has been reported in their study. The results are illustrated in Figure 12D. These results demonstrate the effectiveness of their proposed structure as a sensor. Further, to explore the potential of toroidal EIT based sensors in the multi-band frequency regime, Shen et al. numerically and experimentally reported a dual band toroidal EIT MM in the GHz frequency regime (Shen et al., 2020). The proposed structure consisted of E− ε shaped meta-atoms. The MM was shown to exhibit dual transparency windows having peaks at 7.6 GHz and 10.27 GHz, respectively. The sensing application of their proposed structure was explored. Here as the refractive index of the surrounding media was changed from 1.0 to 1.5 in steps of 0.1, both the transparency peaks got red-shifted. The calculated sensitivities of both the windows were reported as 1.57 GHz/RIU and 2.10 GHz/RIU (Refractive index unit). The efficient use of an EIT based MM as a sensor has been reported in the optical range by Liu et al. (Liu et al., 2010). In their study, they demonstrated a localized surface plasmon resonance sensor by showing a clear shift in the reflectance spectrum via the change of the liquid near to the gold nano-structures in their meta-atom. Water (n = 1.332) and 25 percent aqueous glucose solution (n = 1.372) were used to demonstrate the sensing capacity of the MM. The sensitivity (S) of the nanostructure ensemble was reported to be ≈ 588 nm/RIU. Taking this study into consideration, we investigated the THz toroidal EIT-based sensing capacity of the MM geometry we had designed for a study on toroidal multiband transparency (Bhattacharya et al., 2021). We varied the RI (n) of an analyte layer of thickness 4 μm, coated over the meta-atom, and studied the transmission spectra by varying the refractive index. The refractive index was varied from n = 1.2 to n = 2.0. Figure 12E shows the transmission spectra of the MM for varying n. The black trace indicates transmission for n = 1.2, whereas the red trace indicates the same for n = 1.3. Transmission for RI (n) = 1.4 is shown by the blue curve, whereas that for n = 1.5 is shown by the purple curve. The green trace shows the transmission for n = 1.6. The results indicate that the two transparency peaks show a redshift on the increase of n from 1.2 to 1.6, as is evident from Figure 12E. Further, we presented the relationship between the refractive index and the shift in resonance frequency on increasing n. This is shown in Figure 12F. The black trace shows the frequency shift for the second transmission dip, whereas the red trace shows the corresponding shift for the third transmission dip on an increase of n. We observe a linear shift in the frequency with increase in refractive index. We also calculated the sensitivity (S) of the transmission dips. This is achieved by taking the derivative of frequency shift versus refractive index plot. The sensitivities for second and third transmission dips are 161 GHz/RIU and 135 GHz/RIU (RIU, refractive index unit), respectively. These results reflect the use of our proposed EIT based toroidal MM as a refractive index sensor. Thus, the investigation and exploration of such toroidal-EIT MMs pave a path for the development of new generations of photonic sensors.

### Other applications

Highly sensitive EIT based biosensors have seen considerable interest in the recent past (Yan et al., 2019; Yang et al., 2019). In such studies, a small perturbation of the dielectric environment shifts the narrow transmission profile. Owing to the extremely narrow line-width and high Q factor of toroidal resonances, toroidal MMs provide an excellent platform for the faster and much superior detection of biomolecules and proteins. Experimental detection of Zika virus protein has been reported using toroidal meta-surface by Ahmadivand et al. (Ahmadivand et al., 2017, 2018). Hence, toroidal EIT based MM could find application in the field of biosensing in the near future. The EIT phenomenon in MM could also be significant in the design of ultra-narrow and broad-band absorbers. Numerous absorbers have been reported based on the EIT effect in MMs in the optical (He et al., 2015) and terahertz frequency regime (Yao et al., 2015). Because the toroidal resonance possesses a high Q factor and ultra-narrow bandwidth, such absorbers can be explored based on the toroidal EIT effect.

## Future prospects

Toroidal EIT phenomenon is yet to go a long way, in terms of scientific research as well as technological developments in the design and construction of photonic devices. It is still a trending topic among the MM-based research community. Numerous innovations can be made in the field of toroidal EIT devices in the future. Although the passive modulation of the single and dual-band EIT effect, via the destructive interference between toroidal and electric/magnetic dipolar resonances, has been widely explored, the active tuning is yet to be investigated profoundly. Dynamic modulation of single and multiband transparency windows in graphene-based hybrid toroidal metal structures in the terahertz frequency regime could present ample development potential. Studies made on EIT effects in toroidal resonators have been sensitive to a particular direction of incident light. Polarization insensitive EIT effect in toroidal-dipolar or toroidal-toroidal resonators may lead to sensing and slow light effect, irrespective of the polarization direction of the incident light. Along with experimental studies, significant progress can be made by devising new analytical and theoretical models to explain the toroidal EIT effect. Another aspect that may be explored and taken advantage of is the high Q factor of toroidal resonances. Although toroidal excitation coupled to electric and magnetic resonances has been explored in the context of EIT, the toroidal-toroidal EIT effect may be investigated more keenly. The coupling between toroidal-toroidal resonances resulting in EIT has not been reported in the terahertz frequency regime. This lays down a wide platform to engineer such toroidal coupled EIT devices in the THz spectrum. The coupling of toroidal-toroidal excitation may result in high Q factor EIT windows which will find use in highly sensitive sensors. Further, the spectrum of numerous biological tissues exhibits a toroidal signature. In this context, toroidal excitation-based EIT can provide ample scope in the design of biosensors. The sensing capability of EIT based devices coupled with toroidal resonances could be significant in virus detection, including the Covid-19 virus. The slow light effect in multiband frequency regimes is also yet to be explored more explicitly. Intense research on toroidal based EIT in MMs could bring about crucial and noteworthy innovations in the field of light-matter interaction. Sensing, slow light, and switching application-based devices may see enormous growth in the field of MMs via ardent research on the toroidal EIT phenomenon.

## Conclusions

In this review, we have provided an overview of toroidal resonances and their significance. The identification of toroidal excitations, which is crucial for their application in MMs, has been discussed. We explained how surface current profile, magnetic field profile, and a multipole expansion of electromagnetic moments can be defining factors in comprehending toroidal excitation-based dominance in MM geometry. Further, the phenomenon of EIT has been discussed. We provided a summary of toroidal dipolar-based EIT phenomenon in MMs, showing single and dual-band transparency effects. The EIT phenomenon has been discussed in length by the interference of toroidal and electric or magnetic dipolar excitations. We have also covered the limited work that has so far been done on multiband transparency effect via toroidal coupled resonances. Extensive literature review indicates that toroidal multiband EIT in the terahertz spectrum is yet to be investigated extensively. To address this limited work in the field, we discussed a MM geometry based on toroidal EIT, which successfully demonstrates toroidal multiband EIT in the THz spectrum. The coupling between adjacent resonators has been elaborated using electric field profiles. The electric field profiles show that the strong coupling between the toroidal resonators to the electric dipolar resonators leads to the multiband windows. Further, the transparency windows could be passively modulated by increasing the distance between adjacent resonators. A blue shift in the transparency windows was revealed and further confirmed by theoretical modeling. In this review, we have also addressed the various applications of toroidal EIT, including the slow light phenomenon, biomolecular, and refractive index sensing as well as narrow and broadband absorption. Toroidal-based EIT devices could explore such applications which may contribute remarkably in the field of MMs. Finally, we provide a glimpse into the research that could be pursued in the future in the field of toroidal excitation-based EIT phenomenon in MMs with potential application in the design and development of highly sensitive sensors, tunable modulators, and photonic components.
